# The complete mitochondrion genome of the Hoge’s Side-necked turtle Ranacephala hogei (Chelidae), a critically endangered species from South America

**DOI:** 10.1590/1678-4685-GMB-2024-0203

**Published:** 2025-08-15

**Authors:** Ana Teresa Dumans, Alexandre Pedro Selvatti, Deise Schroder Sarzi, Carolina Furtado, Gláucia Drummond, Marcos Coutinho, Daniel Cardoso Carvalho, Francisco Prosdocimi

**Affiliations:** 1 Universidade Federal do Rio de Janeiro, Instituto de Bioquímica Médica Leopoldo de Meis, Laboratório de Genômica e Biodiversidade, Rio de Janeiro, RJ, Brazil.; 2 Universidade Federal do Estado do Rio de Janeiro (UNIRIO), Centro de Ciências Biológicas e da Saúde, Departamento de Genética e Biologia Molecular, Rio de Janeiro, RJ, Brazil.; 3 Instituto Nacional de Câncer, Departamento de Genética, Rio de Janeiro, RJ, Brazil.; 4 Universidade Federal de Minas Gerais (UFMG), Instituto de Ciências Biológicas, Laboratório de Ecologia e Conservação, Belo Horizonte, MG, Brazil.; 5 Centro Nacional de Pesquisa e Conservação de Répteis e Anfíbios (RAN/ICMBio), Belo Horizonte, MG, Brazil.; 6 Pontifícia Universidade Católica de Minas Gerais (PUC Minas), Programa de Pós-Graduação em Biodiversidade e Meio Ambiente, Laboratório de Genética da Conservação, Belo Horizonte, MG, Brazil.

**Keywords:** Testudines, Pleurodira, mitogenome, endangered chelonians

## Abstract

The Hogei’s side-necked turtle, *Ranacephala hogei*, an endemic species of the Southern Paraíba basin, is the most endangered species of chelonians in Brazil. Here, we sequenced, assembled and described the complete mitogenome for *R. hogei*. The circularized mitogenome of *R. hogei* was 16,513 bp in length, containing all the typical 13 protein-coding genes, 2 rRNAs, 22 tRNAs and the D-loop region, as expected for animal mitogenomes. The gene arrangement also met the expectation for vertebrates, though *R. hogei NAD6* gene was shorter than observed in other closely related species. Additionally, we provided an assembly of the *Podocnemis expansa* mitochondrial genome based on public data in SRA database. Maximum likelihood and Bayesian phylogenomic trees were constructed using the concatenation of the alignments of all protein coding and ribosomal genes and compared to the data obtained from other 15 complete mitochondrial genomes available for the suborder Pleurodira plus five Cryptodira taxa as outgroups in the GenBank database. Our phylogenomic results placed the mitogenome of *R*. *hogei* in a monophyletic South American clade which is deeply within the family Chelidae, corroborating the evolutionary affinities of the sequence. The remaining phylogenomic results also agrees with previous phylogenetic studies in Pleurodira, namely the reciprocal monophyly of the Australasian and South American Chelidae clades, and the monophyly of all higher clades such as families and suborder.

In this study we aim to present the complete sequence of the mitochondrial genome for two turtles, together with a brief analysis and data validation. The Hoge’s side-necked turtle (*Ranacephala hogei;* Mertens, 1967) is a species endemic to the Southern Paraíba basin, and the most endangered species of chelonians in Brazil. Within these countries, it inhabits freshwater habitats such as rivers, streams, ponds, and marshes. Its distribution within these regions varies depending on some main factors such as habitat availability, water quality, and human impact. This turtle is classified among the 25 most endangered freshwater chelonians species in the World (Stanford et al., 2018), and the International Union for Conservation of Nature (IUCN) considers it to be critically endangered (Drummond *et al*., 2022). And *Podocnemis expansa* (Schweigger, 1812) is known as the giant South American turtle, being the largest of the side-neck turtles (Pleurodira) found in the Amazon Basin. Known as the only living turtle that exhibits parental care (Ferrara et al., 2013), *P. expansa* was evaluated as Near Threatened (NT) in the Brazilian territory (Vogt *et al*., 2023) and depends on ongoing conservation efforts, particularly in the rivers where it reproduces.

In the present study, we described for the first time the complete mitochondrial genome of a specimen of *R. hogei* collected in the drainage basin of Southern Paraíba River, located between the states of Minas Gerais and Rio de Janeiro (Brazil). Also, we provided a new assembly for the published mitogenome of *Podocnemis expansa* (MF359933; Wang et al., 2018). Finally, we performed phylogenomic inferences with other Pleurodira species that present their complete mitochondrial genomes available in GenBank to confirm correct placement of clades.

A specimen of *R. hogei* was live captured in a baited funnel trap at the Rio Carangola (20º49’52.29”S, 41° 59’47.35”W) at Paraíba River Basin, in Southeast Brazil. The individual was marked by cutting notches in the carapace and returned to the environment after collecting blood samples. The DNA was extracted from blood using the modified Salting-Out method (Aljanabi and Martinez, 1997). The genome library was prepared using purified DNA with Nextera library kit and run in 1/5 th of a lane on a HiSeq 2500 DNA sequencer (Illumina). MIRA software v. 4.0 (Chevreux et al., 1999) was run using a backbone-guided assembly using the mitochondrial genome of *Chelus fimbriata* (HQ172156; Wang et al., 2012) as reference. This run provided a partial mitogenome assembly that was completed after nine iterations of MITObim v. 1.8 (Hahn et al., 2013). After confirming circularization, an automatic annotation was performed using both the *geneChecker.py* script (Schomaker-Bastos and Prosdocimi, 2018; Allio et al., 2020) and MitosWeb Server (Bernt et al., 2013). The annotations were combined by manual curation using Artemis software 18.2.0 (Carver et al., 2012).

For *P. expansa*, a total of 174.7 million 100 bp reads were downloaded from the Sequence Read Archive (SRA; SRR649422) and split into two equal-sized fastq files using the “*fastq-dump*” from SRAToolkit 2.11.0. A subset of 30 million paired-end reads was randomly subsampled and used as input for *de novo* assembly using MIRA. This run provided a complete version of the mitochondrial genome in two contiguous sequence files when compared to the mitogenome of *Podocnemis unifillis* (JF802204). We used these two contigs to manually produce a version of the mitogenome used as a backbone for a new reference-based assembly by MIRA, this time using the complete set of reads to add coverage, resulting in the complete assembled mitogenome. Automatic annotation was followed by manual curation performed as described for *R. hogei*.

The sequencing of *R. hogei* partial genome produced 59,161,890 reads submitted to the SRA database (SRR28304487). The final mitogenome assembly for *R. hogei* was 16,513 bp in length and had 5,867 reads distributed along the mitogenome with average coverage of 31.5x, according to Tablet software v. 1.17.08.17 (Milne et al., 2012), with GC content of 40.2%. Therefore, one mitochondrial read was found for ~10,000 reads analyzed. The coverage across the assembled mitogenome is detailed in Figure S1. The assembled mitogenome presented 13 protein-coding genes, 22 tRNAs, 2 rRNAs, and one non-coding control region. The annotated sequence was deposited in GenBank under the accession number MF615513.

The final *P. expansa* mitogenome assembly was 16,652 bp in length and had 8,716 reads distributed along the mitogenome, presenting an average read coverage of 52,5x according to MIRA and Tablet, with a GC content of 40.2%. The coverage is detailed in the Figure S2. This mitogenome has the typical constitution expected for vertebrates although the control region was longer than a previous assembly (MF359933), containing 1,142 bp with a 3’ end minisatellite region composed of three 73-75 bp repeats, each with an inserted TA[6-7] dinucleotide microsatellite (Pearse et al., 2006). The annotated sequence was deposited in GenBank under the accession number BK010443.

To confirm the correct placement of these new mitogenomes and revalidate the samples obtained, we downloaded the complete mitochondrial genomes of 15 species of the suborder Pleurodira plus five outgroup species of the sister suborder Cryptodira. The phylogenomic analysis was executed by the concatenation of all protein-coding and ribosomal genes alignments containing *R. hogei*, *P. expansa* and the other species of the suborder Pleurodira and the outgroup species of Cryptodira. To reflect their biological and evolutionary distinctiveness, the alignments of protein-coding and ribosomal genes were conducted through two different strategies. First, the coding sequences were aligned using the translation and reading-frame aware software TranslatorX (Abascal et al., 2010) with the MAFFT 7.5 aligner (Katoh and Standley, 2013). Second, the ribosomal genes were aligned with the Q-INS-I strategy in MAFFT online service (Katoh *et al.,* 2019), which considers the secondary structure of RNA. The resulting alignments were concatenated using the AMAS program (Borowiec, 2016) resulting in the final dataset. For tree building, two different strategies were used. The first tree was built from the concatenated alignment partitioned by gene under the Maximum Likelihood framework, which selected the best substitution models and partition schemes using the program IQ-TREE 2.4 (Minh et al., 2020) and 100 non-parametric bootstrap replicates were used to measure branch support. The second strategy was performed in the Bayesian framework in the MrBayes 3.2.7a (Ronquist et al., 2012), which used the same partition scheme selected by IQ-TREE (eight partitions) and the GTR model plus Γ rate variation for each partition. Parameters remained unlinked for each partition to accommodate the complexity of the evolutionary processes in deep time of the lineages sampled. Four chains of 1,000,000 generations sampled every 1,000th and a 25% burnin were used in the Bayesian phylogenetic reconstruction.

The new assemblies provided here were generated with either sampled material (*R. hogei*) or raw short reads available on the SRA database (*P. expansa*). The analysis showed that the mitochondrial genome of *R. hogei* was similar to the ones previously studied in other species from the same family, unless by a 35-bp shorter *NAD6* gene.

A 16,520 bp complete mitogenome of *P. expansa* has been published earlier (MF359933; Wang et al., 2018), though this previously published sequence lacks some repeat regions found here. Furthermore, the only difference between the two genomes is a 16-bp sequence inside rRNA 12S, that appears twice in the published genome and once in the version described here. Those differences might reflect polymorphisms expect to occur between individuals sampled and sequenced.

Our tree topologies (Figure 1) were consistent with previous turtle phylogenomic studies that sampled a wide variety of molecular markers, including mitochondrial and nuclear data (Pereira et al., 2017; Thomson et al., 2021; Selvatti et al., 2023), confirming the expected placement of the mitogenomes in Testudines major clades. The Maximum Likelihood and Bayesian topologies were identical, and the support for all relationships was very high, with all nodes obtaining 100% of Bayesian posterior probability and most nodes with 100% non-parametric bootstrap. The only two nodes with less than 100% bootstrap support were restricted to the Chelidae clade: (i) the first clade contained *Elseya* sister to *Emydura* and *Myuchelys* with 99% of bootstrap, and the topology was identical to previous phylogenomic studies that included mitochondrial loci (Pereira *et al.,* 2017; Selvatti *et al*., 2023), whereas it was differed from nuclear-only data (Thomson *et al.,* 2021), which recovered *Myuchelys* as the sister to the remaining two genera; (ii) the second and lowest supporting relationship in our study regarded the genera *Elusor* and *Rheodytes*, which formed a clade with 89% bootstrap. This relationship was recovered in studies containing nuclear and mitochondrial (Pereira *et al.,* 2017) and nuclear-only (Thomson *et al.,* 2021) data, but in a mitogenomic-only dataset (Selvatti *et al*., 2023) the genus *Rheodytes* was the sister to a clade containing *Elusor* and one species of *Myuchelys* (*M*. *purvisi*), which rendered the genus *Myuchelys* paraphyletic. Although those results might indicate conflicting phylogenetic signals to resolve, they do not affect the results of this study. For the remaining nodes, the relationships were consistent with previous studies regarding the monophyly of Pleurodira suborder (Georges et al., 1999) and the relationship among its families Chelidae, Pelomedusidae and Podocnemididae with high confidence. The Chelidae clade was split into the clade Chelodininae that was the sister to a monophyletic *Chelodina* genus. Sister to the Australasian clade was the monophyletic South American clade (Chelinae), including *R. hogei, C. frimbriata*, and *Platemys platycephala*. The mitogenome of *R. hogei* clustered with the genus *Platemys*, agreeing with previous genomic studies that indicate an early split of *C. fimbriata* and a more recent divergence of *R. hogei* within Chelinae (Pereira *et al.,* 2017; Thomson *et al.,* 2021; Selvatti *et al*., 2023). Finally, the Podocnemididae family, our second reported mitogenome of *Podocnemis expansa* clustered with its congeneric *P*. *unifilis* forming a sister clade with *Peltocephalus* which, in turn, clustered within the Pelomedusidae family, forming the sister group of Chelidae.

**Figure 1 -:**
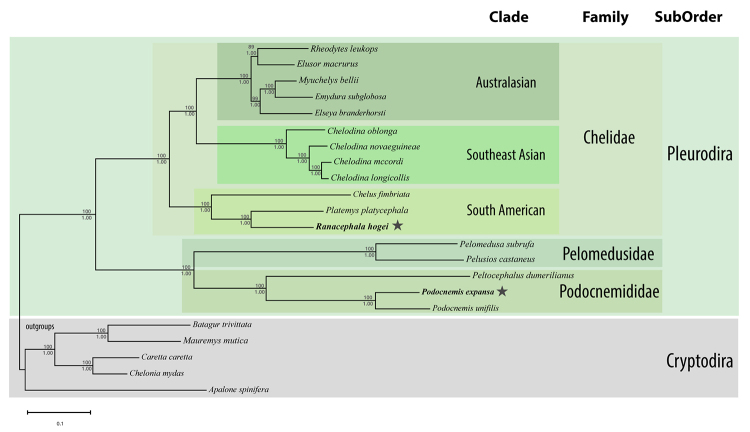
Maximum Likelihood tree based on the concatenation of all protein-coding and ribosomal genes alignments. Numbers show the node bootstrap support values. The species names in bold font represent the mitogenomes described here. GenBank numbers are top to bottom: KY857558, KU736930, KY924930, KC692462, KC692461, KY705234, KY776446, KY705231, KJ713173, HQ172156, KC692464, MF615513, AF039066, KC692463, AB970731, BK010443, JF802204, KX817298, DQ453753, FR694649, AB012104, JF966197. The scale bar indicates the number of substitutions per site.

The current study reported two new assemblies of mitochondrial genomes for two species of Pleurodira turtles, the critically endangered *Ranacephala hogei* and *Podocnemis expansa*. These results increase the genetic information available for those two remarkable freshwater turtle species, and may contribute to future studies in phylogenetics, population genetics, and especially in studies with emphasis on the conservation of these turtle species.

## Data Availability

The genome sequences data that support the findings of this study are openly available in GenBank of NCBI at https://www.ncbi.nlm.nih.gov under the accession numbers MF615513 and BK010443. For *R. hogei,* the associated BioProject, SRA, Study and Bio-Sample numbers are PRJNA1086901, SRR28304487, SRP494752 and SAMN40418757. For *P. expansa,* the associated BioProject, SRA, Study and Bio-Sample numbers are PRJNA177923, SRR649422, SRP016894, and SAMN01886697.

## Supplementary material

The following online material is available for this article:

Figure S1 - Read coverage for the mitogenome sequence of *Ranacephala hogei.*

Figure S2 - Read coverage for the mitogenome sequence of *Podocnemis expansa.*
