# Supplementary material for: The complete mitochondrion genome of the Hoge’s Side-necked turtle Ranacephala hogei (Chelidae), a critically endangered species from South America
Source: Genet Mol Biol. 2025 Aug 15;48(3):e20240203. doi: 10.1590/1678-4685-GMB-2024-0203 (PMC12359128; doi:10.1590/1678-4685-GMB-2024-0203)
Supplement: Figure S1 - [file 1415-4757-GMB-48-03-e20240203-s1.pdf]

Genetics and Molecular Biology

https://doi.org/10.1590/1678-4685-GMB-2024-0203

Supplementary Material to " The complete mitochondrion genome of the Hoge's Side-necked turtle *Ranacephala hogei* (Chelidae), a critically endangered species from South America"

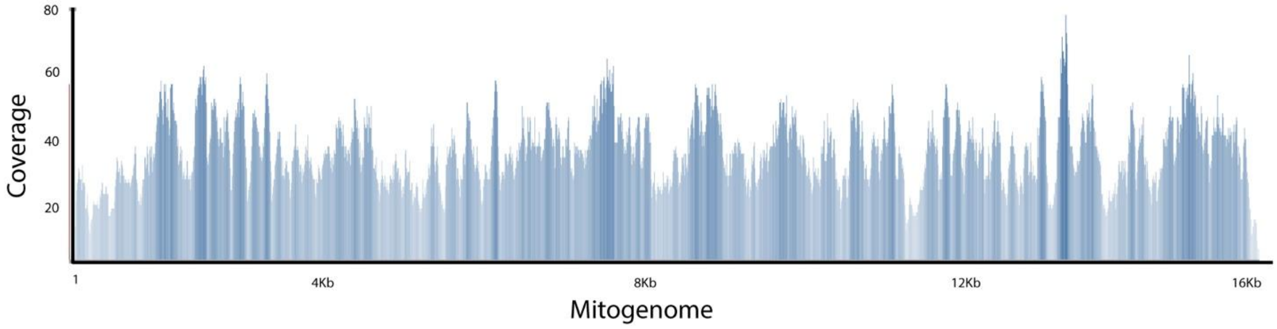

Figure S1 - Read coverage for the mitogenome sequence of *Ranacephala hogei*.
